# Methylation of three genes encoded by X chromosome in blood leukocytes and colorectal cancer risk

**DOI:** 10.1002/cam4.4056

**Published:** 2021-06-18

**Authors:** Tian Tian, Haoran Bi, Ding Zhang, Yupeng Liu, Hongru Sun, Chenyang Jia, Ting Zheng, Hao Huang, Jinming Fu, Lin Zhu, Yashuang Zhao

**Affiliations:** ^1^ Department of Epidemiology Public Health College Harbin Medical University Harbin Heilongjiang The People’s Republic of China

**Keywords:** colorectal cancer, DNA methylation, *FAM156B*, *PIH1D3*, *PPP1R3F*

## Abstract

X chromosome change has been proved to be associated with carcinogenesis and related to gender differences in cancer risk. If aberrant methylation of genes encoded by X chromosome involve in the risk and prognosis of cancers, including colorectal cancer (CRC), remain unclear. We conducted a case–control study consisted of 432 CRC cases and 434 controls, detecting the methylation levels of *FAM156B*, *PIH1D3*, and *PPP1R3F* in the X chromosome in blood leukocytes using methylation‐sensitive high‐resolution melting (MS‐HRM). We analyzed the relationship between the methylation levels and CRC susceptibility and then explored the interactions with environmental factors on CRC risk with logistics regression. Moreover, we conducted a follow‐up study containing 225 CRC patients to explore the associations between the methylation of *FAM156B*, *PPP1R3F*, and *PIH1D3* and CRC prognosis. The hypermethylation of *FAM156B*, *PPP1R3F*, and *PIH1D3* was related to increased CRC risk (OR_PS‐adj_ = 2.932, 95% confidence interval [CI]: 2.029–4.237; OR_PS‐adj_ = 1.602, 95% CI: 1.078–2.382; OR_PS‐adj_ = 1.628, 95% CI: 1.065–2.490, respectively). In the multiple CpG site methylation (MCSM) analysis, compared with non‐MCSM, a significant relationship between MCSM and increased CRC risk was found (OR_PS‐adj_ = 2.202, 95% CI: 1.512–3.208). We observed synergistic interaction between PPP1R3F hypermethylation and fried food consumption on CRC risk (OR_i_ = 2.682, 95% CI: 1.321–5.446). However, there were no associations between the methylation of *FAM156B*, *PPP1R3F*, and *PIH1D3* and CRC prognosis (*p* > 0.05). In conclusion, the methylation of *FAM156B*, *PPP1R3F*, and *PIH1D3* genes in blood leukocytes is significantly related to CRC risk and may be potential biomarkers for CRC risk but not prognosis.

## Graphical abstract

Our results showed that the methylation of *FAM156B*, *PPP1R3F*, and *PIH1D3* genes in blood leukocytes is significantly associated with colorectal cancer (CRC) risk and may be potential biomarkers for CRC risk.

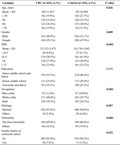

## 1 INTRODUCTION

Colorectal cancer (CRC) is the third most frequent cancer and the second leading cause of cancer death, accounting for more than 1.8 million new cases and an estimated 881,000 deaths all over the world in 2018.^1^ In China, CRC ranks second in terms of incidence and fifth in terms of mortality among all malignant cancers, with an increasing trend in both incidence and mortality.^2^ To date, the overall survival rate of CRC has been increased in Asia. However, the 5‐year survival rate is still about 60%.^3^

Accumulating evidence supports that multiple genetic and epigenetic changes result in CRC.^4^, ^5^ DNA methylation, a crucial player in epigenetic modifications, is essential for development and proper cell functioning. Abnormal CpG islands methylation in gene promoter is associated with many cancers including CRC, which leads to gene silence through hypermethylation or gene activation through hypomethylation and usually occurs in the early stage of cancer development.^6^, ^7^ In addition, abnormal DNA methylation may affect CRC development and prognosis.^8^

Multiple studies focus on the associations between the risk and prognosis of tumors and DNA methylation alterations in tissues. In contrast with tissues, blood sampling is accessible and noninvasive, which makes it more readily to assess tumor risk and prognosis in population‐based studies.^9^, ^10^ In addition, studies reveal that immunologic processes associated with inflammation in tumor progression may affect the leukocyte subpopulations, which may lead to epigenetic changes in peripheral blood. To date, increasing studies focus on DNA methylation as biomarkers for malignancies using peripheral blood leukocytes.^10^, ^11^, ^12^

It has been proved that X chromosome changes correlate with malignancies and are related to gender differences in cancer risk.^13^, ^14^ There are many genes located on the X chromosome playing a vital role in cancers.^15^ *FAM156B* (located at Xp11.22) is one of the transmembrane proteins’ (TMEMs) family. Abnormal methylation of TMEMs is associated with cancer risks, such as lymphomas, gastric carcinoma, and CRC.^16^, ^17^, ^18^ Recent evidence suggests that *PIH1D3*, a protein that identified as an anoikis effector gene, may involve in the progression of tumor.^19^ It has been found that *PIH1D3* emerges as a new player of the cytoplasmic pre‐assembly pathway by stabilizing and promoting both outer and inner dynein arms, loss function of which may cause ciliary and flagella immotility and cause health issues.^20^, ^21^ Previous studies have observed the significant upregulation of *PIH1D3* in human hepatocellular carcinoma and breast cancer tissues.^22^, ^23^ Protein phosphatase 1 (PP1), one of the most highly conserved enzymes involved in cellular processes, consists of the catalytic subunit and regulatory subunit.^24^ *PPP1R3F* (located at Xp11.23) is a major membrane‐associated glycogen targeting subunit of PP1, regulating glycogen synthase in astrocytoma cells.^25^ Studies have observed the abnormal accumulation of glycogen in tumor cell lines,^26^, ^27^ indicating the possible association between *PPP1R3F* and cancer risk. To date, the research about the associations between *FAM156B*, *PIH1D3*, and *PPP1R3F* and cancer mainly focus on the gene expression and conducted on tumor tissue and cell lines, the relationship between methylation of *FAM156B*, *PIH1D3*, and *PPP1R3F* and cancer risk and prognosis is still unclear.

Our research was conducted to explore the relationship between the methylation of *FAM156B*, *PIH1D3*, and *PPP1R3F* in white blood cells (WBC) and CRC risk. Moreover, genetic susceptibility plays a crucial part in CRC etiology, which may interact with environmental factors.^28^, ^29^ We explored the interactions between genes methylation status and environmental factors on CRC risk in WBC. We also prospectively followed up a cohort of CRC patients to estimate the associations between methylation of *FAM156B*, *PIH1D3*, and *PPP1R3F* and CRC prognosis in China.

## 2 METHODS

### 2.1 Study population

The subjects consisted of 432 primary CRC patients which were diagnosed with pathology and underwent surgery at the Cancer Hospital of Harbin Medical University and the Second Affiliated Hospital of Harbin Medical University from 1 November 2004 to 1 May 2010 and 434 cancer‐free controls collected from the Second Affiliated Hospital of Harbin Medical University during the same period. Patients with neuroendocrine carcinoma, malignant melanoma, non‐Hodgkin's lymphoma, gastrointestinal stromal tumors, and Lynch syndrome were excluded. In addition, according to the self‐report, we excluded the controls with a history of gastrointestinal disease. In the follow‐up study, a total of 225 from the 434 primary CRC were obtained from November 2004 to March 2014 by hospital records or telephone interview. Written informed consent from each subject was obtained, and our research was conducted in light of the Declaration of Helsinki. The study was approved by the Research Ethics Committee of Harbin Medical University.

All the subjects completed the structured questionnaires interviewed by well‐trained interviewers. The information of the structured questionnaires included demographic characteristics, lifestyles, dietary status during the past 12 months preceding the interview. We also collected data about CRC family history. Moreover, clinical information was collected from medical records including the information of tumor size, histological and pathological types, and the level of serum carcinoembryonic antigen (CEA) and carbohydrate antigen 19‐9 (CA19‐9) before surgery. During follow‐up, we obtained the treatment protocol of patients and the information of disease progression, recurrence, and the date and cause of death. Survival time was the period from the first diagnosis of CRC to the time of any cause of death or the end of follow‐up.

### 2.2 DNA extraction and bisulfite conversion

Genomic DNA was isolated using the QIAamp DNA Blood Mini kit (Qiagen, Hilden, Germany) from whole blood. We measured the DNA quantity and quality using NanoDrop 2000 (Thermo Scientific, Waltham, MA, USA). Moreover, DNA bisulfite‐modified was carried out using the EpiTect Plus DNA Bisulfite Kit (Qiagen).

### 2.3 Methylation‐sensitive high‐resolution melting analysis

We used methylation‐sensitive high‐resolution melting (MS‐HRM) analysis to detect the gene methylation status on the LightCycler480 machine (Roche Applied Science, Mannheim, Germany). Primer sequences, reaction systems, and amplification conditions of the three genes for MS‐HRM analysis are summarized in Table S1. MS‐HRM data were analyzed by the software module of Gene Scanning (Roche Applied Science, version 2.0). We mixed the commercially available 100% methylated and 0% methylated human whole genomic DNA to construct methylated DNA standards as standard curves, including 100%, 25%, 10%, 5%, 2%, 1%, and 0% methylated DNA (Zymo Research Corp., Irvine, CA, USA) (Figure S1 **)**. Moreover, the blank and repeated experiment were performed in our study.

### 2.4 Statistical analysis

Student's *t*‐test and Chi‐squared test were used to evaluate the homogeneity between CRC patients and controls. Receiver operating characteristic curve and cut‐off value determined by the Youden index method were conducted to categorize all subjects into hypomethylation group and hypermethylation group.^30^, ^31^ We estimated the associations between FAM156B, PIH1D3, and PPP1R3F methylation and CRC risk with corresponding odds ratios (ORs) and 95% confidence intervals (95% CIs) by unconditional univariate and multivariate logistic regression analyses. Propensity score (PS) analysis was applied to control confounding factors. Multivariate logistic regression model was applied for estimating the PS, including the possible variables which were related to CRC risk (Table S2). The authenticity and stability of our results were detected using PS adjusting methods and PS matching analysis. We applied crossover analyses to evaluate gene–environment interaction effects on CRC risk. To explore the gene–gene interaction on CRC risk, multifactor dimensionality reduction (MDR) analysis was applied. Life table method and log‐rank test were performed to estimate survival rate and compare the different survival rates. Cox proportional hazard regression analysis was applied for assessing the associations between FAM156B, PIH1D3, and PPP1R3F methylation and CRC prognosis with corresponding hazard ratio (HR) and 95% CI. We constructed the survival curve using Kaplan–Meier. The missing values were filled by multiple imputations.^32^, ^33^ All statistical tests were two‐sided. *p* values <0.05 and *p* values <0.025 were considered significant in the overall analysis and subgroup analysis by Bonferroni correction. The statistical analyses were conducted using SPSS Statistics version 24.0 (IBM, Inc., Armonk, NY, USA) and MDR software version 3.0.1 (Unix, San Antonio, TX, USA).

## 3 RESULTS

### 3.1 Characteristics of subjects

The basic characteristics of the subjects are summarized in Table 1 and Table S2. The mean age (±standard deviation) was 60 (±11.457) years for patients and 58 (±10.994) years for controls. About 60.9% of cases and 52.1% of controls were men. The mean body mass index (BMI) was 23.212 kg/m^2^ for patients and 24.170 kg/m^2^ for controls. The distributions of age, gender, BMI, occupation, marital status, ethnic group, and family history of CRC were significantly different between CRC patients and controls, which were adjusted in the following analyses.

**TABLE 1:** Characteristics of colorectal cancer patients and control

Variables	CRC (*n* = 432), *n* (%)	Control (*n* = 434), *n* (%)	*p* value*
Age (years), mean ± SD	60 ± 11.457	58 ± 10.994	**0.026**
≤50	86 (19.9%)	103 (23.7%)	
50–	138 (32.0%)	144 (33.2%)	
60–	122 (28.2%)	133 (30.6%)	
>70	86 (19.9%)	54 (12.5%)	
Gender			**0.009**
Male	263 (60.9%)	226 (52.1%)	
Female	169 (39.1%)	208 (47.9%)	
BMI, mean ± SD	23.212 ± 3.473	24.170 ± 3.892	**<0.001**
<18.5	38 (8.8%)	27 (6.1%)	
18.5–	218 (50.5%)	181 (41.8%)	
24–	120 (27.8%)	125 (28.9%)	
≥27	56 (12.9%)	101 (23.2%)	
Education			0.059
Junior middle school and below	239 (s55.3%)	210 (48.4%)	
Senior middle school	111 (25.6%)	115 (26.4%)	
University and above	82 (19.1%)	109 (25.2%)	
Occupation			**<0.001**
Blue collar	52 (11.9%)	87 (20.0%)	
White collar	211 (48.8%)	242 (55.7%)	
Both	169 (39.3%)	105 (24.3%)	
Marriage			**0.007**
Married	422 (97.6%)	408 (94.0%)	
Others	10 (2.4%)	26 (6.0%)	
Nationality			**<0.001**
The Han nationality	388 (89.8%)	349 (80.4%)	
Others	44 (10.2%)	85 (19.6%)	
Family history of colorectal cancer			**0.022**
No	402 (93.0%)	419 (96.5%)	
Yes	30 (7.0%)	15 (3.5%)	

Bold values indicate *p* value < 0.05 was considered statistically significant.

Abbreviations: BMI, body mass index; CRC, colorectal cancer; SD, standard deviation.

* *p* value calculated using Student's *t*‐test for continuous variables or Pearson's chi‐squared test for categorical variables for overall data and using the paired *t*‐test or McNemar's test for matched paired data.

### 3.2 Methylation of *FAM156B*, *PIH1D3*, and *PPP1R3F* and CRC risk

After adjusting for the demographic confounding factors, hypermethylation of *FAM156B*, *PPP1R3F*, and *PIH1D3* was associated with increased CRC risk (Table 2). Consistent results were shown in PS adjustment analysis (OR_PS‐adj_ = 2.932, 95% CI: 2.029–4.237, *p* < 0.001; OR_PS‐adj_ = 1.602, 95% CI: 1.078–2.382, *p* = 0.021; OR_PS‐adj_ = 1.628, 95% CI: 1.065–2.490, *p* = 0.025, respectively). In PS matching analysis, we successfully matched 227 patients and observed that there was a significant difference between the hypermethylation of *FAM156B* and CRC risk (OR_PS‐paired_ = 1.687, 95% CI: 1.290–2.208, *p* < 0.001) (Table S3).

**TABLE 2:** The relationship between methylation of individual genes and colorectal cancer risk before and after propensity score adjustment

Genes^a^		CRC (%)	Controls (%)	OR_adj_ ^b^	95% CI	*p* value*	OR_PS‐adj_ ^c^	95% CI	*p* value*
*FAM156B*	Hypomethylation	202 (46.8%)	291 (67.0%)	1.000			1.000		
	Hypermethylation	230 (53.2%)	143 (33.0%)	5.130	3.334–7.895	<0.001	2.932	2.029–4.237	<0.001
*PPP1R3F*	Hypomethylation	163 (37.8%)	184 (42.5%)	1.000			1.000		
	Hypermethylation	269 (62.2%)	250 (57.5%)	1.850	1.230–2.783	0.004	1.602	1.078–2.382	0.021
*PIH1D3*	Hypomethylation	310 (71.7%)	347 (80.0%)	1.000			1.000		
	Hypermethylation	122 (28.3%)	87 (20.0%)	1.877	1.283–2.748	0.001	1.628	1.065–2.490	0.025
*MCSM*	Non‐MCSM	107 (24.8%)	157 (36.2%)	1.000			1.000		
	MCSM‐L	117 (27.1%)	118 (27.2%)	2.229	1.418–3.504	0.001	1.682	1.018–2.780	0.043
	MCSM‐H	208 (48.1%)	159 (36.6%)	5.032	3.100–8.170	<0.001	2.633	1.579–3.942	<0.001
	MCSM	325 (75.2%)	277 (63.8%)	2.992	1.985–4.510	<0.001	2.202	1.512–3.208	<0.001

Abbreviations: BMI, body mass index; CI, confidence interval; CRC, colorectal cancer; MCSM, multiple CpG site methylation; OR, odds ratio; PS, propensity score; ROC, receiver operating characteristic.

^a^ The cutoffs of individual genes determined by the ROC curve were: *FAM156B*: 2%; *PPP1R3F*: 2%; *PIH1D3*: 10%.

^b^ ORs adjusted for age, gender, BMI, occupation, marital status, nationality, and family history of CRC.

^c^ PS adjusted OR means adjusted for PS as a covariate.

* *p* value < 0.05 was considered statistically significant.

### 3.3 Multiple CpG site methylation and CRC risk

We divided multiple CpG site methylation (MCSM) of multiple genes into four types: none of the genes methylated was identified as non‐MCSM; MCSM‐L was defined as two genes methylated; MCSM‐H was identified as more than two genes methylated; MCSM was defined as one and more genes methylated.

In our study, compared with non‐MCSM, there were significant associations between MCSM, MCSM‐Ln and MCSM‐H and CRC risk after PS adjustment (OR_PS‐adj_ = 2.202, 95% CI: 1.512–3.208, *p* < 0.001; OR_PS‐adj_ = 1.682, 95% CI: 1.018–2.780, *p* = 0.043; OR_PS‐adj_ = 2.633, 95% CI: 1.579–3.942, respectively) (Table 2).

### 3.4 Subgroup analysis

We carried out several subgroup analyses based on age (≤60 and >60 years), gender, tumor location, and tumor Duck's stage (Duck's stage A + B and C + D).

In subgroup analysis stratified by gender, we observed significant associations between *FAM156B* hypermethylation and CRC risk in males and females (OR_PS‐adj_ = 4.110, 95% CI: 2.193–7.701; OR_PS‐adj_ = 6.604, 95% CI: 3.049–14.305, respectively). Moreover, a significant association between *PPP1R3F* hypermethylation and increased CRC risk in males was observed (OR_PS‐adj_ = 2.301, 95% CI: 1.379–3.840) (Table 3). The results stratified by age showed that the patients <60 years with the hypermethylation of *FAM156B* and *PIH1D3* were related to increased CRC risk (OR_PS‐adj_ = 3.873, 95% CI: 2.295–6.536; OR_PS‐adj_ = 2.209, 95% CI: 1.074–4.542). Moreover, the associations between the hypermethylation of *FAM156B*, *PIH1D3*, and *PPP1R3F* and CRC risk in patients with different tumor locations and tumor Duck's stages are shown in Table S4.

**TABLE 3:** The associations between methylation of the genes and colorectal cancer risk in subgroup analyses

		Gender											
		Male						Female					
DNA methylation		Case (%)	Control (%)	OR_adj_ (95% CI)	*p* value*	OR_PS‐adj_ (95% CI)	*p* value*	Case (%)	Control (%)	OR_adj_ (95% CI）	*p* value*	OR_PS‐adj_ (95% CI)	*p* value*
*FAM156B*	Hypo‐	187 (71.1%)	201 (88.9%)					15 (8.9%)	90 (43.3%)				
	Hyper‐	76 (28.9%)	25 (11.1%)	3.549 (2.063–6.106)	<0.001	4.110 (2.193–7.701)	<0.001	154 (91.1%)	118 (56.7%)	8.978 (4.415–18.255)	<0.001	6.604 (3.049–14.305)	<0.001
*PPP1R3F*	Hypo‐	150 (57.0%)	163 (72.1%)					13 (7.7%)	21 (10.1%)				
	Hyper‐	113 (43.0%)	63 (27.9%)	2.089 (1.358–3.213)	0.001	2.301 (1.379–3.840)	0.002	156 (92.3%)	187 (89.9%)	1.142 (0.508–2.569)	0.747	1.032 (0.319–3.343)	0.956
*PIH1D3*	Hypo‐	225 (85.6%)	199 (88.0%)					85 (50.3%)	148 (71.2%)				
	Hyper‐	38 (14.4%)	27 (12.0%)	1.368 (0.638–2.935)	0.402	1.249 (0.512–3.052)	0.607	84 (49.7%)	60 (28.8%)	2.316 (1.345–3.988)	0.003	2.101 (0.931–4.743)	0.071
*MCSM*	Non‐MCSM	101 (38.4%)	147 (65.0%)					6 (3.6%)	10 (4.8%)				
	MCSM‐L	106 (40.3%)	50 (21.7%)	3.302 (2.707–4.028)	<0.001	3.164 (2.532–3.954)	<0.001	11 (6.5%)	68 (32.7%)	0.271 (0.148–0.496)	<0.001	0.196 (0.101–0.379)	<0.001
	MCSM‐H	56 (21.3%)	29 (13.3%)	2.912 (2.288–3.706)	<0.001	3.448 (2.629–4.521)	<0.001	152 (89.9%)	130 (62.5%)	1.829 (1.059–3.156)	0.03	1.140 (0.633–2.052)	0.663
	MCSM	162 (61.6%)	79 (35.0%)	3.156 (2.651–3.756)	<0.001	3.264 (2.682–3.973)	<0.001	163 (96.4%)	198 (95.2%)	1.246 (0.726–2.141)	0.425	0.808 (0.449–1.456)	0.479

DNA methylation		Age											
		≤ 60						>60					
		Case (%)	Control (%)	OR_adj_ (95% CI)	*p* value*	OR_PS‐adj_ (95% CI)	*P* value*	Case (%)	Control (%)	OR_adj_ (95% CI)	*p* value*	OR_PS‐adj_ (95% CI)	*p* value*
*FAM156B*	Hypo‐	99 (44.8%)	178 (72.1%)					103 (48.8%)	113 (60.4%)				
	Hyper‐	122 (55.2%)	69 (27.9%)	7.642 (4.215–13.856)	<0.001	3.873 (2.295–6.536)	<0.001	108 (51.2%)	74 (39.6%)	3.404 (1.856–6.242)	<0.001	2.141 (1.218–3.764)	<0.001
*PPP1R3F*	Hypo‐	82 (37.1%)	107 (43.3%)					81 (38.4%)	77 (41.2%)				
	Hyper‐	139 (62.9%)	140 (56.7%)	1.885 (1.101–3.227)	0.022	1.596 (0.956–2.666)	0.074	130 (61.6%)	110 (58.8%)	1.921 (1.037–3.557)	0.038	1.601 (0.943–2.719)	0.081
*PIH1D3*	Hypo‐	155 (69.7%)	206 (83.4%)					155 (73.5%)	141 (75.4%)				
	Hyper‐	66 (30.3%)	41 (16.6%)	2.570 (1.320–5.003)	0.008	2.209 (1.074–4.542)	0.033	56 (26.5%)	46 (24.6%)	1.378 (0.644–2.945)	0.383	1.159 (0.492–2.729)	0.715
*MCSM*	Non‐MCSM	51 (23.1%)	93 (37.7%)					56 (26.5%)	64 (34.2%)				
	MCSM‐L	59 (26.7%)	78 (31.5%)	2.188 (1.725–2.776)	<0.001	1.746 (1.340–2.276)	<0.001	58 (27.5%)	40 (21.4%)	2.409 (1.826–3.177)	<0.001	1.755 (1.311–2.350)	<0.001
	MCSM‐H	111 (50.2%)	76 (30.8%)	7.155 (5.347–9.468)	<0.001	3.340 (2.672–4.404)	<0.001	97 (45.0%)	83 (44.4%)	3.303 (2.419–4.509)	<0.001	1.924 (1.490–2.484)	<0.001
	MCSM	170 (76.9%)	154 (62.3%)	3.271 (2.620–4.084)	<0.001	2.569 (2.053–3.215)	<0.001	155 (72.5%)	123 (65.8%)	2.719 (2.105–3.512)	<0.001	1.861 (1.471–2.355)	<0.001

Abbreviations: CI, confidence interval; MCSM, multiple CpG site methylation; OR, odds ratio; PS, propensity score.

* *p* < 0.025 was considered statistically significant.

Stratified by gender, significant associations between MCSM‐L, MCSM‐H, and MCSM and increased CRC risk in males and significant relationship between MCSM‐L and decreased CRC risk in females were observed. Moreover, stratified by age, tumor location, and Duck's stage, we observed that individuals carrying MCSM, MCSM‐L, and MCSM‐H had increased CRC risk in all stratified groups (Table 3).

### 3.5 Interactions and combination effects between gene methylation and environmental factors on CRC risk

As shown in Table 4, significant synergistic interaction between *PPP1R3F* hypermethylation and intake of fried food >1 time/month on CRC risk was found (OR_i_ = 2.682, 95% CI: 1.321–5.446, *p* = 0.006). In addition, significant combination effects between gene methylation and environmental factors on CRC risk are shown in Table S5.

**TABLE 4:** Combinations and interactions of genes methylation and environmental factors on colorectal cancer risk

	Hypo‐	Hyper‐	Interaction	
	OR_eg_ (95% CI)^a^		OR_i_ (95% CI)^a^	*p* value*
*FAM156B*				
Roughage (g/week)				
>200	1.000	0.703 (0.41–1.206)		
≤200	5.608 (3.587–8.766)	2.429 (1.271–4.640)	1.988 (0.953–4.148)	0.067
Physical exercise				
Yes	1.000	0.990 (0.646–1.519)		
No	3.887 (2.293–6.588)	7.007 (4.104–12.18)	1.837 (0.963–3.505)	0.065
*PPP1R3F*				
Fried food (time/month)				
≤1	1.000	1.452 (0.925–2.278)		
>1	0.999 (0.593–1.682)	3.889 (2.135–7.085)	**2.682 (1.321–5.446)**	**0.006**

* *p* < 0.05 was considered statistically significant. Bold values indicate statistically significant *p* value and corresponding 95% Confidence Interval.

Abbreviations: BMI, body mass index; CI, confidence interval; OR, odds ratio.

^a^ Adjusted by age, gender, BMI, marriage, nationality, occupation, family history of CRC.

### 3.6 Gene–gene interaction and CRC risk

We explored the gene–gene interactions between the methylation of *FAM156B*, *PIH1D3* and *PPP1R3F* on CRC risk using MDR. The association of gene–gene higher order interaction on CRC risk is shown in Table 5. The MDR model with the best testing accuracy included the methylation of *FAM156B*, *PIH1D3*, and *PPP1R3F* (testing accuracy = 0.610) with a maximum testing accuracy of 61.01% and a maximum cross‐validation consistency of 10 out of 10 followed by statistical significance of 1000‐fold permutation test (*p* < 0.01).

**TABLE 5:** Gene–gene interactions of *FAM156B*, *PIH1D3*, and *PPP1R3F* methylation on the risk of colorectal cancer analyzed by the multifactor dimensionality reduction method

Model	Training Bal. Acc. (%)	Testing Bal. Acc. (%)	*p* value*	Cross‐validation consistency
*FAM156B*	0.601	0.601	10 (0.001)	10/10
*FAM156B*, *PPP1R3F*	0.601	0.601	10 (0.001)	10/10
*FAM156B*, *PIH1D3*, *PPP1R3F*	0.613	0.610	10 (0.001)	10/10

* *p* value < 0.05 was considered statistically significant.

### 3.7 The associations between *FAM156B*, *PIH1D3*, and *PPP1R3F* methylation and CRC prognosis

A total of 225 patients completed the follow‐up. The median follow‐up time is 66 months. The pathological type of CRC, preoperative CA19‐9, and CEA level, application of anastomat in surgery and Duke's stage were adjusted in the analysis (Table S6).

There was no significant relationship between *FAM156B*, *PIH1D3*, and *PPP1R3F* methylation and CRC prognosis (Table 6). Moreover, in the subgroup analyses, we did not find significant associations between the methylation of *FAM156B*, *PIH1D3*, and *PPP1R3F* and CRC prognosis (Tables S7–S9).

**TABLE 6:** Association between gene methylation and colorectal cancer survival

Genes^a^	No. of CRC (%)	Median survival time (month)	3‐Year survival (%)	5‐Year survival (%)	Univariate analyses		Multivariate analyses		PS analyses	
					HR (95% CI)	*p* value*	HR_ad_ ^b^ (95% CI)	*p* value*	HR_ps‐adj_ ^c^ (95% CI)	*p* value*
*FAM156B*										
Hypomethylation	202 (89.8%)	72.0 (2.9)	66	57	1.000		1.000		1.000	
Hypermethylation	23 (10.2%)	63.2 (4.8)	72	58	0.848 (0.426–1.686)	0.600	1.063 (0.522–2.165)	0.866	1.257 (0.627–2.522)	0.520
*PPP1R3F*										
Hypomethylation	88 (39.1%)	68.2 (4.2)	64	54	1.000		1.000		1.000	
Hypermethylation	137 (60.9%)	75.9 (3.6)	68	60	0.738 (0.499–1.091)	0.100	0.897 (0.589–1.365)	0.611	1.174 (0.764–1.803)	0.460
*PIH1D3*										
Hypomethylation	190 (84.4%)	73.0 (3.0)	67	58	1.000		1.000		1.000	
Hypermethylation	35 (15.6%)	68.6 (6.5)	62	52	1.105 (0.647–1.889)	0.700	1.301 (0.742–2.281)	0.358	1.004 (0.581–1.734)	0.990

Abbreviations: CA19‐9, carbohydrate antigen 19‐9; CEA, carcinoembryonic antigen; CI, confidence interval; CRC, colorectal cancer; HR, hazard ratio; ROC, receiver operating characteristic.

^a^ The cutoffs of each gene determined by ROC curve were: *FAM156B*: 5%, *PPP1R3F*: 0%, *PIH1D3*: 10%.

^b^ HR_adj_ for age, gender, Duke's Stage, anastomat on surgery, and preoperative CA19‐9 level in multivariate Cox regression analysis.

^c^ HR_PS‐adj_ adjusted for PS as a covariate in multi‐Cox analysis including age, gender, pathological type, Duke's Stage, anastomat on surgery, tumor size, preoperative CA19‐9 level, and CEA level.

* *p* < 0.05 was considered statistically significant.

## 4 DISCUSSION

In our research, we evaluated the relationship between the methylation of *FAM156B*, *PIH1D3*, and *PPP1R3F* and CRC risk and prognosis for the first time.

Serving as one of the TMEMs, *FAM156A* encodes a transmembrane protein, which molecular function annotated by the gene ontology terms relates to gene protein binding and methylated histone binding. Our results suggested that hypermethylation of 22 CpGs between the first exon and first intron of the *FAM156B* gene in WBC is most associated with increased CRC risk. Members of *TMEMs* have been observed abnormal expression in many cancers, such as the downregulation of *TMEM106A* resulted from the hypermethylation of promoter region in GC cell lines^34^ and abnormal expression of *TMEM176A* and *176B* in breast, lymph, skin, and liver cancer.^16^ Moreover, a previous study observed significant deregulated expression of *TMEMs* in clear cell renal cell carcinoma tumors, suggesting that *TMEMs* may be a descriptor of the most advanced tumors.^35^ As for the important role of the *TMEM* family in cancer progression, the positive association between *FAM156B* methylation and CRC risk may imply the effect of *FAM156B* in CRC pathology.

*PPP1R3F* is characterized as one of the PP1 catalytic subunits. As an important eukaryotic protein serine/threonine phosphatase, PP1 regulates various cellular functions by interacting with the regulatory subunits. Studies observed that glycogen metabolism is essential for tumor cell pathophysiology and abnormal glycogen metabolism has been found in many tumor cells including CRC.^26^, ^27^, ^36^ As a critical part of glycogen metabolism, dephosphorylation of glycogen synthase is catalyzed by PP1 bound to *PPP1R3*. Studies revealed that family proteins of *PPP1R3* play an essential role in recruiting PP1 to glycogen and increasing the specific activity of PP1 toward specific glycogen synthase.^24^, ^37^, ^38^ As a member of *PPP1R3* family proteins, *PPP3RF* was found to be important to neuronal activities.^25^ Our results about the association between *PPP1R3F* hypermethylation and CRC risk may propose the effect of *PPP1R3F* in the carcinogenic progress. We also observed significant synergistic interaction between *PPP1R3F* hypermethylation and intake of fried food (>1 time/month) on CRC risk. The consumption of fried food was related to the kinds of tumors. Several carcinogenic substances during the fried food cooking, particularly heterocyclic aromatic compounds and acrylamide carcinogen, could alter DNA structure and cause DNA methylation alterations.^39^, ^40^

*PIH1D3*, as one of the PIH1 protein family, located on chromosome Xq22.3, interacts with heat shock protein 90 (Hsp90).^41^ Studies indicated that PIH1 proteins exert influence in axonemal dyneins and assembly and preribosomal RNA processing. Moreover, *PIH1D3* has been regarded as an anoikis gene in a study conducted to identify novel anoikis effector genes through genome‐wide screening.^19^ Anoikis is defined as apoptosis caused by cell detachment from the extracellular matrix or inappropriate cell–matrix interactions.^42^ Anoikis involves in tissue homeostasis, development, and oncogenic processes, breakdown of which may contribute to neoplasia.^42^ In our research, we observed the hypermethylation of *PIH1D3* was associated with CRC risk, which may result from the loss function of *PIH1D3* caused by methylation changes.

The PS method was used to reduce the bias in estimating effects and offer investigators the ability to reduce the likelihood of confounding when analyzing the observational data. Of note, it was revealed that hypermethylation of the three genes was still significantly associated with increased CRC risk with smaller OR and narrower confidence intervals. The results made our conclusions of significant associations between the hypermethylation of *FAM156B*, *PIH1D3*, and *PPP1R3F* and CRC risk more robust and reliable.

Subgroup analysis in our study showed the different relationships between methylation levels of *FAM156B*, *PIH1D3*, and *PPP1R3F* and CRC risk in different genders. Hypermethylation of *FAM156B* was significantly related to increased CRC risk. The estimated effects in females were twice as high as men. Whether in the cases and controls, the proportion of *FAM156B* hypermethylation in females was higher than that in males (cases: 91.1% vs. 28.9%; controls: 56.7% vs. 11.1%). Gender effects for methylation loci on the X chromosome mostly resulted in the X‐inactivation dosage compensation mechanism in females.^43^ X chromosome in females achieved dose compensation by silencing one X chromosome, which is named X chromosome inactivation.^44^ Studies revealed that anti‐oncogenes that escape from X‐inactivation resulted in cancer bias. Moreover, hypermethylation was related to the transcriptional silencing of many X‐linked loci on the inactive X chromosome.^45^ Our research revealed the gender‐bias DNA methylation in the X chromosome in CRC and better clarified the effect of the methylation of the genes in CRC development in females.

In addition, we analyzed the datasets of GEO (GSE51032) to corroborate our results.^46^ The average methylation of 13 probes annotated to *PIH1D3* and 17 probes annotated to *PPP1R3F* was evaluated. We analyzed the probe located on *PIH1D3* (cg07896193), which was in the differentially methylated region (DMR) detected in our research. Due to the DMR in our study was located in the gene CpG islands, we analyzed the average methylation level of 12 probes located in CpG islands annotated to *PPP1R3F*. We also detected the associations by the quintile or median distributions of *PIH1D3* and *PPP1R3F* methylation levels in the GSE51032 to evaluate the results’ stability. We observed a marginally statistical significance between the methylation of *PPP1R3F* at CpG island and CRC risk (OR_adj_ = 2.043, 95% CI: 0.971–4.297, *p* = 0.060), as well as the marginal association between *PIH1D3* hypermethylation at a single CG site (cg 07896193) (OR_adj_ = 1.716, 95% CI: 0.984–2.933, *p* = 0.057) (Table 7). In subgroup analysis stratified by gender and age, we only found the association between *PPP1R3F* hypermethylation and decreased CRC risk in older than 60 in the GEO dataset. The different results between our study and the external dataset may be explained by the different CG detection sites and different methods to detect methylation levels (Tables S10 and S11).

**TABLE 7:** Associations between *PPP1R3F* methylation and the colorectal cancer risk in the validation dataset

DNA methylation^a^		Case (%) (*n* = 166)	Control (%) (*n* = 424)	OR_adj_ ^b^ (95% CI)	*p* value*
*PPP1R3F*	Hypomethylation	96 (57.8%)	140 (33.0%)	1.000	
	Hypermethylation	70 (42.2%)	284 (67.0%)	1.245 (0.647–2.396)	0.513
*PPP1R3F‐CpG islands*	Hypomethylation	92 (55.4%)	144 (34.0%)	1.000	
	Hypermethylation	74 (44.6%)	280 (66.0%)	**2.043 (0.971–4.297)**	**0.060**
*PIH1D3*	Hypomethylation	113 (68.1%)	182 (42.9%)	1.000	
	Hypermethylation	53 (31.9%)	242 (57.1%)	0.758 (0.459–1.253)	0.280
*PIH1D3*‐cg07896193	Hypomethylation	102 (61.4%)	193 (45.6%)	1.000	
	Hypermethylation	64 (38.6%)	230 (54.4%)	**1.716 (0.98–2.993)**	**0.057**

Bold values indicate statistically significant *p* value and corresponding 95% CI.

Abbreviations: CI confidence interval; OR odds ratio.

^a^ The cutoff value for *PPP1R3F* was set according to the second quintile of the gene methylation level; the cutoff value for *PIHID3* was set according to the median of the gene methylation level.

^b^ The ORs were adjusted by age and gender.

* *p* < 0.05 was considered statistically significant.

Our results did not observe the associations between methylation levels of *FAM156B*, *PIH1D3*, and *PPP1R3F* and CRC prognosis. To date, there are no studies to explore the relationship between the methylation of *FAM156B*, *PIH1D3*, and *PPP1R3F* and CRC prognosis.

There are still some limitations to our research. Firstly, recall bias cannot be avoided due to the case–control study based in the hospital. Second, the sample size in the follow‐up study is small, which may limit the statistical power in our research.

In conclusion, we observed significant associations between the hypermethylation of *FAM156B*, *PIH1D3*, and *PPP1R3F* and CRC risk in WBC. It suggested that the methylation levels of these three genes in WBC might be the predictive biomarkers for identifying high‐risk individuals who can develop into CRC. Moreover, gene–environment interaction may play a vital role in CRC risk. However, methylation of *FAM156B*, *PIH1D3*, and *PPP1R3F* might not serve as potential biomarkers for CRC survival.

## CONFLICT OF INTEREST

The authors declare that there is no conflict of interest that could be perceived as prejudicing the impartiality of this review.

## AUTHORS’ CONTRIBUTIONS

L.Z. and Y.Z. designed the study, directed its implementation, including quality assurance and control, and reviewed the manuscript. T.T., H.B., and D.Z. put forward and helped the study's analytic strategy. T.T. wrote the manuscript, and T.T., H.B., and Y.L. did the data analysis. H.S., C.J., T.Z., H.H., and J.F. performed the experimental work and contributed to the sample collection. The work described has not been submitted elsewhere for publication, in whole or in part. All the authors have reviewed the final version of the manuscript and approved it for publication.

## ETHICAL APPROVAL STATEMENT

Written informed consent from all participants was obtained. The study was conducted in accordance with the declaration of Helsinki and approved by the Research Ethics Committee of Harbin Medical University.

## Supporting information

Supplementary Material

## Data Availability

The data generated or analyzed during the present study are available from the corresponding author on reasonable request.
