# Supplementary material for: Methylation of three genes encoded by X chromosome in blood leukocytes and colorectal cancer risk
Source: Cancer Med. 2021 Jun 18;10(14):4964–76. doi: 10.1002/cam4.4056 (PMC8290255; doi:10.1002/cam4.4056)
Supplement: Supplementary file 1 — Supplementary Material [file CAM4-10-4964-s001.docx]

**Figure S1. Methylation-sensitive high-resolution melting (MS-HRM) for WBC *FAM156B, PIH1D3 and PPP1R3F* methylation standard samples.**

***FAM156B***


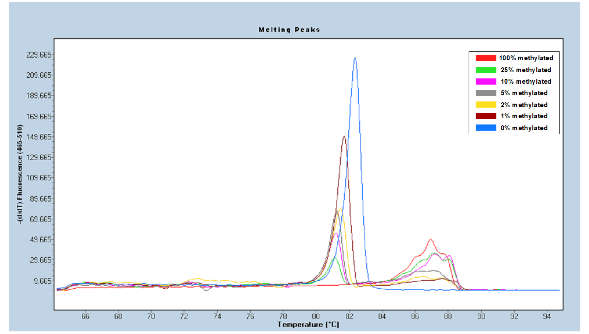

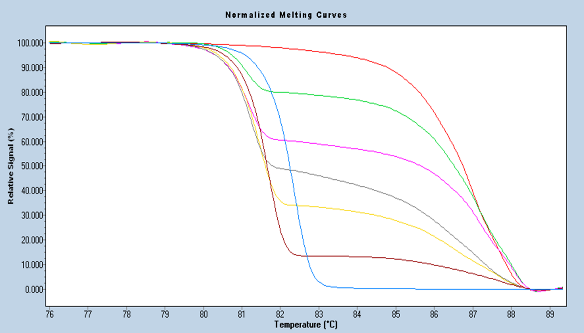


a1 b1

***PIH1D3***


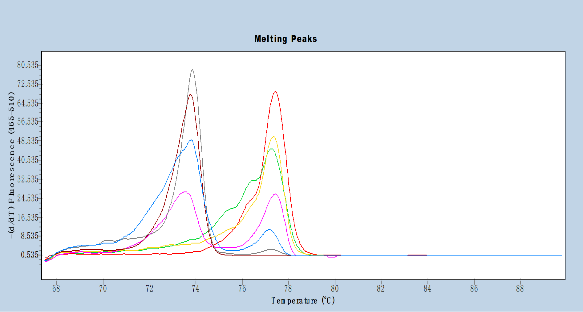

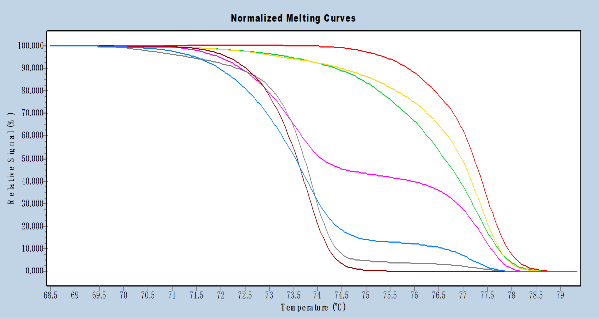


a2 b2

***PPP1R3F***


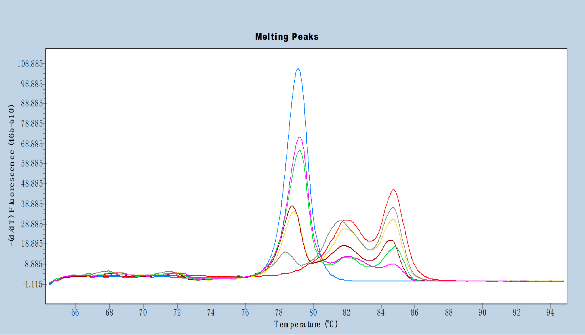

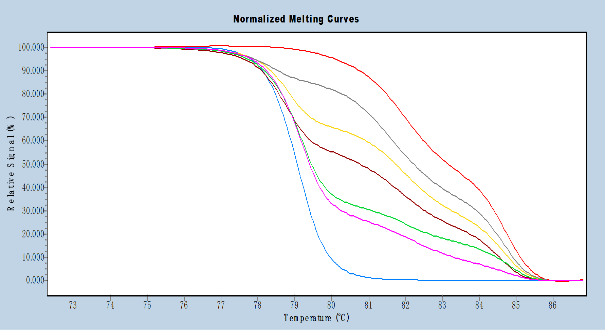


a3 b3

Note: a1, a2, a3: Standard curves for MS-HRM assay of *FAM156B, PIH1D3* and *PPP1R3F*; b1, b2, b3: Fluorescence profile obtained at the melting temperature for serial dilutions of methylated DNA (100%, 50%, 25%, 10%, and 5%, 2%, 1% and 0%) from each gene

| **Table S1. Primer sequences for the genes.** | |  |  |  |  |
| --- | --- | --- | --- | --- | --- |
| **Gene** | **Primer sequence（5'-3'）** | **Ensemble gene ID** | **Gene location（GRCh38/hg38）** | **Sense / Antisense complementary strand** | **Amplified fragment length (bp)** |
| *FAM156B* | F：GTTGCGTTTTTAGGGTAGAGGAAA | ENSG00000179304 | chrX:52897383-52908560 | Sense | 22/140 |
|  | R：CCCGCTCTCAATCTCTCC |  |  |  |  |
| *PPP1R3F* | F：AGGAAGAGGAGGTTTGTTT | ENSG00000049769 | chrX:49267843-49288095 | Sense | 14/142 |
|  | R：GCCTCCAACTCCACCATA |  |  |  |  |
| *PIH1D3* | F：GGGAAAAGCGTAAAAGAGA | ENSG00000080572 | chrX:107204632-107244243 | Sense | 12/145 |
|  | R：CCCTCTAACGTCTAACAATC |  |  |  |  |

| **Table S2. Distribution of characteristics of colorectal cancer patients and controls before and after propensity score adjustment.** | | | | | | |
| --- | --- | --- | --- | --- | --- | --- |
| Variables | **Overall** | | | **Propensity score matched** | | |
|  |  |  |  |  |  |  |
|  | **CRC**  **(n=432), n (%)** | **Control**  **(n=434), n (%)** | ***P* value^*^** | **CRC**  **(n=227), n (%)** | **Control**  **(n=227), n (%)** | ***P* value^*^** |
| Age, years |  |  | **0.026** |  |  | 0.971 |
| Mean ± SD | 60±11.457 | 58±10.994 |  | 58±11.613 | 58±20.29 |  |
| ≤ 50 | 86 (19.907%) | 103 (23.733%) |  | 50 (22.026%) | 52 (22.907%) |  |
| 50- | 138 (32.037%) | 144 (33.18%) |  | 78 (34.361%) | 76 (33.480%) |  |
| 60- | 122 (28.241%) | 133 (30.645%) |  | 66 (29.079%) | 68 (29.956%) |  |
| >70 | 86 (19.815%) | 54 (12.442%) |  | 33 (14.537%) | 31 (13.656%) |  |
| Gender |  |  | **0.009** |  |  | 0.904 |
| Male | 263 (60.880%) | 226 (52.074%) |  | 135 (59.471%) | 134 (58.943%) |  |
| Female | 169 (39.120%) | 208 (47.926%) |  | 92 (40.529%) | 93 (41.101%) |  |
| BMI |  |  | **<0.001** |  |  | 0.498 |
| Mean ± SD | 23.212±3.473 | 24.17±3.892 |  | 23.552±3.711 | 23.506±8.294 |  |
| <18.5 | 38 (8.657%) | 27 (6.129%) |  | 15 (6.740%) | 19 (8.370%) |  |
| 18.5- | 218 (50.556%) | 181 (41.751%) |  | 107 (47.269%) | 109 (47.797%) |  |
| 24- | 120 (27.778%) | 125 (28.894%) |  | 74 (32.511%) | 61 (27.004%) |  |
| ≥ 27 | 56 (13.009%) | 101 (23.226%) |  | 31 (13.568%) | 38 (16.872%) |  |
| Education |  |  | 0.059 |  |  | 0.876 |
| Junior middle school and below | 239 (55.278%) | 210 (48.387%) |  | 123 (54.185%) | 123 (54.185%) |  |
| Senior middle school | 111 (25.648%) | 115 (26.452%) |  | 59 (25.991%) | 55 (24.361%) |  |
| University and above | 82 (19.074%) | 109 (25.161%) |  | 45 (19.824%) | 49 (21.498%) |  |
| Occupation |  |  | **<0.001** |  |  | 0.236 |
| Blue collar | 52 (11.944%) | 87 (20%) |  | 122 (53.656%) | 118 (51.982%) |  |
| White collar | 211 (48.75%) | 242 (55.714%) |  | 29 (12.907%) | 42 (18.502%) |  |
| Both | 169 (39.306%) | 105 (24.286%) |  | 76 (33.48%) | 67 (29.515%) |  |
| Marriage |  |  | **0.007** |  |  | 0.942 |
| Married | 422 (97.639%) | 408 (93.963%) |  | 223 (98.37%) | 223 (98.238%) |  |
| Others | 10 (2.361%) | 26 (6.037%) |  | 4 (1.674%) | 4 (1.762%) |  |
| Nationality |  |  | **<0.001** |  |  | 0.695 |
| The Han nationality | 388 (89.815%) | 349 (80.415%) |  | 194 (85.463%) | 191 (84.141%) |  |
| Others | 44 (10.185%) | 85 (19.585%) |  | 33 (14.537%) | 36 (15.859%) |  |
| Family history of colorectal cancer |  |  | **0.022** |  |  | 0.981 |
| No | 402 (92.963%) | 419 (96.452%) |  | 218 (96.167%) | 218 (96.167%) |  |
| Yes | 30 (7.037%) | 15 (3.548%) |  | 9 (3.877%) | 9 (3.877%) |  |
| Refined grains, g/day |  |  | **<0.001** |  |  | 0.666 |
| ≤ 500 | 378 (87.454%) | 420 (96.774%) |  | 212 (93.392%) | 214 (94.405%) |  |
| > 500 | 54 (12.546%) | 14 (3.226%) |  | 15 (6.608%) | 13 (5.639%) |  |
| Roughage, g/week |  |  | **0.001** |  |  | 0.462 |
| ≤ 200 | 371 (85.926%) | 336 (77.373%) |  | 189 (83.260%) | 183 (80.617%) |  |
| > 200 | 61 (14.074%) | 98 (22.627%) |  | 38 (16.740%) | 44 (19.383%) |  |
| Vegetable, g/day |  |  | **0.034** |  |  | 0.501 |
| ≤ 500 | 279 (64.583%) | 250 (57.558%) |  | 141 (62.115%) | 134 (59.031%) |  |
| > 500 | 153 (35.417%) | 184 (42.442%) |  | 86 (37.885%) | 93 (40.969%) |  |
| Fruit, times/week |  |  | **0.021** |  |  | 0.821 |
| ≤ 7 | 310 (71.713%) | 280 (64.378%) |  | 158 (69.515%) | 155 (68.282%) |  |
| > 7 | 122 (28.287%) | 154 (35.622%) |  | 69 (30.529%) | 72 (31.718%) |  |
| Fat meat |  |  | **<0.001** |  |  | 0.833 |
| No | 204 (47.269%) | 266 (61.198%) |  | 120 (52.996%) | 118 (51.982%) |  |
| Yes | 228 (52.731%) | 168 (38.802%) |  | 107 (47.048%) | 109 (48.018%) |  |
| Pork, kg/week |  |  | **<0.001** |  |  | 0.598 |
| ≤ 1 | 376 (87.083%) | 411 (94.654%) |  | 205 (90.308%) | 208 (91.762%) |  |
| > 1 | 56 (12.917%) | 23 (5.346%) |  | 22 (9.692%) | 19 (8.282%) |  |
| Beef, g/week |  |  | 0.054 |  |  | 0.510 |
| ≤ 250 | 365 (84.444%) | 345 (79.401%) |  | 187 (82.291%) | 181 (79.868%) |  |
| > 250 | 67 (15.556%) | 89 (20.599%) |  | 40 (17.753%) | 46 (20.176%) |  |
| Braised fish , times/week |  |  | **0.001** |  |  | 0.423 |
| ≤ 1 | 276 (63.935%) | 322 (74.101%) |  | 149 (65.771%) | 157 (69.295%) |  |
| > 1 | 156 (36.065%) | 112 (25.899%) |  | 78 (34.273%) | 70 (30.749%) |  |
| Red bean and mung bean |  |  | **0.021** |  |  | 0.739 |
| < 1 | 260 (60.185%) | 227 (52.396%) |  | 131 (57.841%) | 135 (59.383%) |  |
| ≥ 1 | 172 (39.815%) | 207 (47.604%) |  | 96 (42.203%) | 92 (40.661%) |  |
| Category of dairy products |  |  | **<0.001** |  |  | 0.730 |
| Milk only | 299 (69.259%) | 238 (54.931%) |  | 149 (65.771%) | 146 (64.229%) |  |
| Milk and others(Yogurt, coconut milk, milk powder) | 133 (30.741%) | 196 (45.069%) |  | 78 (34.273%) | 81 (35.815%) |  |
| Fried food, times/month |  |  | **0.001** |  |  | 0.710 |
| ≤ 1 | 303 (70.093%) | 346 (79.724%) |  | 165 (72.687%) | 168 (74.009%) |  |
| > 1 | 129 (29.907%) | 88 (20.276%) |  | 62 (27.313%) | 59 (25.991%) |  |
| Leftovers, times/week |  |  | **<0.001** |  |  | 0.864 |
| ≤ 3 | 255 (59.12%) | 312 (71.889%) |  | 147 (64.758%) | 149 (65.771%) |  |
| > 3 | 177 (40.88%) | 122 (28.111%) |  | 80 (35.242%) | 78 (34.273%) |  |
| Physical exercise |  |  | **0.004** |  |  | 0.537 |
| Yes | 268 (62.037%) | 310 (71.382%) |  | 146 (64.317%) | 153 (67.313%) |  |
| No | 164 (37.963%) | 124 (28.618%) |  | 81 (35.683%) | 74 (32.731%) |  |
| Stairs climbing, times/day |  |  | **<0.001** |  |  | 0.250 |
| ≤ 5 | 134 (31.019%) | 30 (6.912%) |  | 38 (16.74%) | 29 (12.907%) |  |
| > 5 | 298 (68.981%) | 404 (93.088%) |  | 189 (83.26%) | 198 (87.137%) |  |
| Smoke |  |  | **0.008** |  |  | 0.656 |
| No | 205 (47.407%) | 245 (56.452%) |  | 107 (47.269%) | 112 (49.339%) |  |
| Yes | 227 (52.593%) | 189 (43.548%) |  | 120 (52.775%) | 115 (50.661%) |  |
| Drink |  |  | **<0.001** |  |  | 0.992 |
| No | 211 (48.796%) | 302 (69.677%) |  | 129 (56.74%) | 129 (56.74%) |  |
| Yes | 221 (51.204%) | 132 (30.323%) |  | 98 (43.304%) | 98 (43.304%) |  |
| CRC, Colorectal Cancer; S.D., standard deviation; BMI, Body Mass Index; | | | | | | |
| ^*^*P* value calculated using Student’s t-test for continuous variables or Pearson’s chi-squared test for categorical variables for overall data and using the paired t-test or McNemar’s test for matched paired data. | | | | | | |

**Table-S3. Association between methylation of individual genes and the risk of CRC after PS-paired analysis.**

| **Genes**^†^ | | **CRC (%)** | **Controls (%)** | **OR_Ps-paired_**^‡^ | **95%CI** | ***P* value^*^** |
| --- | --- | --- | --- | --- | --- | --- |
| *FAM156B* | Hypomethylation | 100 (44.1%) | 160 (70.3%) | 1.000 |  |  |
|  | Hypermethylation | 127 (55.9%) | 67 (29.7%) | 1.687 | **1.290-2.208** | **<0.001** |
| *PPP1R3F* | Hypomethylation | 87 (38.5%) | 111 (49.0%) | 1.000 |  |  |
|  | Hypermethylation | 140 (61.5%) | 116 (51.0%) | 1.245 | 0.91-1.704 | 0.167 |
| *PIH1D3* | Hypomethylation | 165 (72.8%) | 183 (80.4%) | 1.000 |  |  |
|  | Hypermethylation | 62 (27.2%) | 44 (19.6%) | 1.219 | 0.822-1.807 | 0.307 |
| MCSM | Non-MCSM | 57（25.1%） | 96（42.3%） | 1.000 |  |  |
|  | MCSM-L | 56（24.7%） | 60（26.4%） | 1.403 | **1.161-1.695** | **<0.001** |
|  | MCSM-H | 114（50.2%） | 71（31.3%） | 2.047 | **1.725-2.429** | **<0.001** |
|  | MCSM | 170（74.9%） | 131（57.7%） | 1.756 | **1.503-2.052** | **<0.001** |

OR, odds ratio; CI, confidence interval; PS, propensity score;

^*^*P* value＜0.05 was considered statistically significant.

^†^ The cutoffs of individual genes determined by ROC curve were: FAM156B: 2%; PPP1R3F: 2%; PIH1D3: 10%.

^‡^PS paired OR means adjusted for PS matching.

**Table S4. The associations between methylation of the genes and CRC risk in subgroup analyses.**

| **DNA Methylation** | | **Tumor location** | | | | | | | | | | | |
| --- | --- | --- | --- | --- | --- | --- | --- | --- | --- | --- | --- | --- | --- |
|  |  | **Colon** | | | | | | **Rectal** | | | | | |
|  |  | **Case (%)** | **Control(%)** | **OR_adj_（95% CI）** | ***P* value^*^** | **OR_PS-adj_（95% CI）** | ***P* value^*^** | **Case (%)** | **Control(%)** | **OR_adj_（95% CI）** | ***P* value^*^** | **OR_PS-adj_（95% CI）** | ***P* value^*^** |
|  |  |  |  |  |  |  |  |  |  |  |  |  |  |
| FAM156B | hypo- | 118(45.7%) | 291(67.0%) |  |  |  |  | 84(48.3%) | 291(67.0%) |  |  |  |  |
|  | hyper- | 140(54.3%) | 143(33.0%) | 5.560(3.296-9.377) | <0.001 | 3.013(1.972-4.603) | <0.001 | 90(51.7%) | 143(33.0%) | 4.523(2.647-7.728) | <0.001 | 2.914(1.728-4.913) | <0.001 |
| PPP1R3F | hypo- | 96(37.2%) | 184(42.4%) |  |  |  |  | 67(38.5%) | 184(42.4%) |  |  |  |  |
|  | hyper- | 162(62.8%) | 250(57.6%) | 2.009(1.257-3.211) | 0.004 | 1.703(1.093-2.652) | 0.019 | 107(61.5%) | 250(57.6%) | 1.798(1.025-3.154) | 0.041 | 1.737(1.025-2.942) | 0.04 |
| PIH1D3 | hypo- | 187(72.5%) | 347(80.0%) |  |  |  |  | 123(70.7%) | 347(80.0%) |  |  |  |  |
|  | hyper- | 71(27.5%) | 87(20.0%) | 1.920(1.191-3.096) | 0.008 | 1.679(1.001-2.818) | 0.05 | 51(29.3%) | 87(20.0%) | 1.775(1.109-2.840) | 0.017 | 1.588(0.954-2.643) | 0.075 |
| *MCSM* | Non-MCSM | 63(24.5%) | 157(36.2%) |  |  |  |  | 43(24.6%) | 157(36.2%) |  |  |  |  |
|  | MCSM-L | 70(27.2%) | 118(27.2%) | 2.249(1.824-2.773) | <0.001 | 1.837(1.471-2.295) | <0.001 | 49(28%) | 118(27.2%) | 2.503(1.974-3.174) | <0.001 | 2.024(1.563-2.623） | <0.001 |
|  | MCSM-H | 124(48.2%) | 159(36.6%) | 5.072(3.996-6.437) | <0.001 | 2.768(2.261-3.390 | <0.001 | 83(47.4%) | 159(36.6%) | 4.475(3.442-5.817) | <0.001 | 2.772(2.185-3.516） | <0.001 |
|  | MCSM | 194(75.5%) | 227(52.3%) | 3.046(2.510-3.695) | <0.001 | 2.351(1.954-2.829 | <0.001 | 66(75.4%) | 227(52.3%) | 3.121(2.505-3.888) | <0.001 | 2.437(1.962-3.028） | <0.001 |
| **DNA Methylation** | | **Duck's stage** | | | | | | | | | | | |
|  |  | **Duck's stage A+B** | | | | | | **Duck's stage C+D** | | | | | |
|  |  | **Case (%)** | **Control(%)** | **OR_adj_（95% CI）** | ***P* value^*^** | **OR_PS-adj_（95% CI）** | ***P* value^*^** | **Case (%)** | **Control(%)** | **OR_adj_（95% CI）** | ***P* value^*^** | **OR_PS-adj_（95% CI）** | ***P* value^*^** |
|  |  |  |  |  |  |  |  |  |  |  |  |  |  |
| FAM156B | hypo- | 127(50.4%) | 291(67.0%) |  |  |  |  | 75(41.4%) | 291(67.0%) |  |  |  |  |
|  | hyper- | 125(49.6%) | 143(33.0%) | 4.422(2.621-7.459) | <0.001 | 2.618(1.643-4.170) | <0.001 | 105(58.6%) | 143(33.0%) | 5.984(3.509-10.204) | <0.001 | 3.772(2.359-6.031) | <0.001 |
| PPP1R3F | hypo- | 98(38.9%) | 184(42.4%) |  |  |  |  | 65(36.3%) | 184(42.4%) |  |  |  |  |
|  | hyper- | 154(61.1%) | 250(57.6%) | 1.981(1.249-3.142) | 0.004 | 1.549(1.003-2.392) | 0.049 | 115(63.7%) | 250(57.6%) | 1.863(1.092-3.177) | 0.023 | 1.919(1.126-3.272) | 0.018 |
| PIH1D3 | hypo- | 181(71.8%) | 347(80.0%) |  |  |  |  | 129(71.9%) | 347(80.0%) |  |  |  |  |
|  | hyper- | 71(28.2%) | 87(20.0%) | 2.015(1.312-3.096) | 0.001 | 1.584(1.009-2.488) | 0.046 | 51(28.1%) | 87(20.0%) | 1.699(0.927-3.115) | 0.083 | 1.771(0.909-3.452) | 0.089 |
| *MCSM* | Non-MCSM | 68(27.0%) | 157(36.2%) |  |  |  |  | 39(21.7%) | 157(36.2%) |  |  |  |  |
|  | MCSM-L | 70(27.8%) | 118(27.2%) | 2.235(1.819-2.747) | <0.001 | 1.616(1.296-2.014) | <0.001 | 47(26.1%) | 118(27.2%) | 2.531(1.985-3.229) | <0.001 | 2.446(1.880-3.184) | <0.001 |
|  | MCSM-H | 114(45.2%) | 159(36.6%) | 4.281(3.394-5.401) | <0.001 | 2.303(1.881-2.820) | <0.001 | 94(52.2%) | 159(36.6%) | 5.685(4.340-7.446) | <0.001 | 3.682(2.897-4.680) | <0.001 |
|  | MCSM | 184(73.0%) | 227(52.3%) | 2.861(2.367-3.460) | <0.001 | 1.990(1.657-2.389) | <0.001 | 140(78.3%) | 227(52.3%) | 3.425(2.734-4.291) | <0.001 | 3.140(2.516-3.920) | <0.001 |

^*^*P* value＜0.025 was considered statistically significant.

**Table-S5. Combinations and interactions of genes methylation and environmental factors on CRC risk.**

| **Factor** | ***FAM156B*** | | **Interaction** | | ***PPP1R3F*** | | **Interaction** | | ***PIH1D3*** | | **Interaction** | |
| --- | --- | --- | --- | --- | --- | --- | --- | --- | --- | --- | --- | --- |
|  | **Hypo**- | **Hype-** | **OR_i_ (95% CI)**^†^ | ***P* value***** | **Hypo-** | **Hyper-** | **OR_i_ (95% CI)**^†^ | ***P* value***** | **Hypo-** | **Hyper-** | **OR_i_ (95% CI)**^†^ | ***P* value***** |
|  | **OReg (95% CI)**^†^ | |  |  | **OR_eg_ (95% CI)**^†^ | |  |  | **OR_eg_ (95% CI)**^†^ | |  |  |
| Refined grains (g/day) | |  |  |  |  |  |  |  |  |  |  |  |
| ≤ 500 | 1.000 | 3.598 (1.682-7.700) |  | 0.879 | 1.000 | 1.826 (1.196-2.788) |  |  | 1.000 | 1.889 (1.243-2.870) |  |  |
| > 500 | 5.015 (3.234-7.776) | 20.263 (5.556-73.895) | 1.123 (0.251-5.016) |  | 3.231 (1.426-7.319) | 9.278 (3.271-26.314) | 1.573 (0.420-5.894) | 0.502 | 4.050 (1.855-8.840) | 5.944 (0.892-39.591) | 0.777 (0.074-8.192) | 0.822 |
| Roughage (g/week) | |  |  |  |  |  |  |  |  |  |  |  |
| > 200 | 1.000 | 0.703 (0.41-1.206) |  | 0.067 | 1.000 | 1.716 (0.804-3.662) |  |  | 1.000 | 1.751 (0.528-5.804) |  |  |
| ≤ 200 | 5.608 (3.587-8.766) | 2.429 (1.271-4.640) | 1.988 (0.953-4.148) |  | 1.626 (0.887-2.981) | 2.934 (1.587-5.425) | 1.052 (0.480-2.306) | 0.900 | 1.690 (1.032-2.766) | 3.172 (1.857-5.418) | 1.073 (0.262-4.394) | 0.915 |
| Vegetable, g/day | |  |  |  |  |  |  |  |  |  |  |  |
| > 500 | 1.000 | 0.699 (0.465-1.052) |  | 0.155 | 1.000 | 2.168 (1.132-4.151) |  |  | 1.000 | 2.561 (1.326-4.946) |  |  |
| ≤ 500 | 4.448 (2.707-7.307) | 4.426 (2.521-7.771) | 0.653 (0.363-1.175) |  | 1.540 (0.945-2.510) | 2.572 (1.498-4.415) | 0.770 (0.392-1.514) | 0.444 | 1.453 (1.029-2.051) | 2.254 (1.369-3.710) | 0.606 (0.263-1.394) | 0.232 |
| Fruit (times/week) | |  |  |  |  |  |  |  |  |  |  |  |
| > 7 | 1.000 | 0.706 (0.440-1.132) |  | 0.362 | 1.000 | 1.235 (0.670-2.275) |  |  | 1.000 | 2.231 (1.146-4.342) |  |  |
| ≤ 7 | 5.348 (3.258-8.778) | 3.357 (1.885-5.978) | 1.321 (0.727-2.400) |  | 1.041 (0.611-1.775) | 2.251 (1.284-3.945) | 1.750 (0.907-3.380) | 0.095 | 1.526 (1.036-2.247) | 2.579 (1.606-4.141) | 0.758 (0.350-1.641) | 0.480 |
| Fat meat | |  |  |  |  |  |  |  |  |  |  |  |
| No | 1.000 | 1.588 (1.033-2.44) |  | 0.288 | 1.000 | 1.597 (0.926-2.752) |  |  | 1.000 | 1.689 (0.997-2.863) |  |  |
| Yes | 4.396 (2.682-7.206) | 9.936 (5.738-17.203) | 1.423 (0.741-2.733) |  | 1.561 (0.946-2.574) | 3.322 (1.904-5.796) | 1.333 (0.673-2.642) | 0.405 | 1.724 (1.167-2.547) | 3.646 (2.017-6.589) | 1.252 (0.476-3.289) | 0.631 |
| Pork (kg/week) | |  |  |  |  |  |  |  |  |  |  |  |
| ≤ 1 | 1.000 | 2.66 (1.377-5.141) |  | 0.265 | 1.000 | 1.753 (1.127-2.727) |  |  | 1.000 | 1.766 (1.184-2.634) | - |  |
| > 1 | 4.931 (3.152-7.714) | 32.871 (7.351-146.991) | 2.506 (0.499-12.593) |  | 2.210 (1.048-4.661) | 8.742 (3.116-24.522) | 2.257 (0.658-7.741) | 0.194 | 2.507 (1.322-4.754) | - | - | 0.999 |
| Braised fish, times/week | |  |  |  |  |  |  |  |  |  |  |  |
| ≤ 1 | 1.000 | 1.783 (1.141-2.786) |  | 0.581 | 1.000 | 2.174 (1.406-3.363) |  |  | 1.000 | 1.981 (1.222-3.211) |  |  |
| > 1 | 5.409 (3.343-8.753) | 8.027 (4.333-14.872) | 0.832 (0.433-1.598) |  | 2.192 (1.306-3.679) | 3.074 (1.798-5.256) | 0.645 (0.336-1.236) | 0.186 | 1.722 (1.072-2.766) | 2.790 (1.434-5.431) | 0.818 (0.305-2.195) | 0.676 |
| Red bean mung bean, times/week | |  |  |  |  |  |  |  |  |  |  |  |
| < 1 | 1.000 | 5.121 (3.019-8.688) |  | 0.963 | 1.000 | 1.830 (1.038-3.228) |  |  | 1.000 | 2.026 (1.142-3.592) |  |  |
| ≥ 1 | 0.767(0.513-1.149) | 3.874 (2.296-6.539) | 0.986 (0.532-1.825) |  | 1.329 (0.838-2.108) | 2.473 (1.481-4.131) | 1.017 (0.541-1.911) | 0.959 | 1.379 (0.971-1.957) | 2.434 (1.327-4.463) | 0.871 (0.323-2.352) | 0.771 |
| Category of dairy products | |  |  |  |  |  |  |  |  |  |  |  |
| Milk only |  | 5.212 (2.993-9.078) |  | 0.905 | 1.000 | 1.763 (0.908-3.425) |  |  | 1.000 | 1.428 (0.800-2.548) | 1.000 |  |
| Milk and others^‡^ | 0.588 (0.231-1.496) | 2.906 (1.547-5.457) | 0.949 (0.376-2.392) |  | 1.666 (0.664-4.182) | 3.309 (1.687-6.492) | 1.126 (0.496-2.561) | 0.766 | 1.576 (0.899-2.764) | 3.658 (1.901-7.041) | 1.625 (0.698-3.784) | 0.252 |
| Fried food (time/month) | |  |  |  |  |  |  |  |  |  |  |  |
| ≤ 1 | 1.000 | 1.716 (1.109-2.655) |  | 0.679 | 1.000 | 1.452 (0.925-2.278) |  |  | 1.000 | 1.924 (1.255-2.948) | 1.000 |  |
| > 1 | 5.121 (3.199-8.198) | 10.295 (5.344-19.833) | 1.171 (0.553-2.483) |  | 0.999 (0.593-1.682) | 3.889 (2.135-7.085) | **2.682 (1.321-5.446)** | **0.006** | 1.721 (1.113-2.66) | 2.992 (1.413-6.335) | 0.904 (0.322-2.536) | 0.842 |
| Overnight food (times/week) | |  |  |  |  |  |  |  |  |  |  |  |
| ≤ 3 | 1.000 | 1.757 (1.152-2.679) |  | 0.240 | 1.000 | 1.560 (1.004-2.423) |  |  | 1.000 | 1.655 (1.048-2.614) | 1.000 |  |
| > 3 | 4.618 (2.800-7.614) | 12.018 (6.757-21.376) | 1.481 (0.77-2.852) |  | 1.435 (0.885-2.325) | 4.002 (2.234-7.170) | 1.788 (0.905-3.532) | 0.094 | 1.821 (1.274-2.602) | 4.398 (2.100-9.214) | 1.459 (0.600-3.551) | 0.394 |
| Physical exercise | |  |  |  |  |  |  |  |  |  |  |  |
| Yes | 1.000 | 0.99 (0.646-1.519) |  | 0.065 | 1.000 | 1.869 (1.195-2.922) |  |  | 1.000 | 1.890 (1.243-2.874) |  |  |
| No | 3.887 (2.293-6.588) | 7.07 (4.104-12.18) | 1.837 (0.963-3.505) |  | 2.244 (1.294-3.891) | 4.846 (2.513-9.343) | 1.156 (0.604-2.210) | 0.662 | 2.339 (1.385-3.952) | 6.084 (2.875-12.872) | 1.376 (0.539-3.513) | 0.496 |
| Stairs climbing, times/day | |  |  |  |  |  |  |  |  |  |  |  |
| > 5 | 1.000 | 2.662 (1.504-4.71) |  | 0.441 | 1.000 | 1.959 (1.262-3.040) |  |  | 1.000 | 1.860 (1.198-2.887) |  |  |
| ≤ 5 | 4.244 (2.395-7.523) | 13.969 (7.87-24.796) | 0.712 (0.299-1.691) |  | 5.761 (2.873-11.553) | 10.486 (5.389-20.404) | 0.929 (0.366-2.361) | 0.877 | 5.458 (3.238-9.200) | 9.989 (2.827-35.295) | 0.984 (0.222-4.362) | 0.982 |
| Smoking | |  |  |  |  |  |  |  |  |  |  |  |
| No | 1.000 | 2.113 (1.192-3.745) |  | 0.170 | 1.000 | 1.326 (0.763-2.304) |  |  | 1.000 | 1.598 (0.893-2.860) |  |  |
| Yes | 4.647 (2.809-7.688) | 17.409 (8.244-36.765) | 1.773 (0.773-4.069) |  | 0.958 (0.573-1.602) | 2.306 (1.281-4.152) | 1.815 (0.960-3.431) | 0.066 | 1.191 (0.757-1.872) | 2.748 (1.493-5.060) | 1.444 (0.485-4.302) | 0.477 |
| Drinking | |  |  |  |  |  |  |  |  |  |  |  |
| No | 1.000 | 0.168 (0.091-0.31) |  | 0.463 | 1.000 | 1.623 (1.050-2.510) |  |  | 1.000 | 1.586 (0.818-3.074) |  |  |
| Yes | 3.712 (1.481-9.300) | 0.878 (0.441-1.749) | 1.408 (0.565-3.513) |  | 2.584 (1.443-4.628) | 6.067 (3.574-10.299) | 1.446 (0.722-2.895) | 0.296 | 3.041 (1.911-4.837) | 5.744 (3.298-10.004) | 1.191 (0.471-3.010) | 0.700 |

CRC, Colorectal Cancer; BMI, Body Mass Index. ^†^adjusted by age, gender, BMI, marriage, nationality, occupation, family history of CRC. ^‡^Yogurt, coconut milk, milk powder. ^*^*P* value＜0.05 was considered statistically significant.

**Table-S6 Association between clinical parameters and CRC survival.**

| **Characteristic** | **CRC (n=225), n (%)** | **Median survival time (month) (SD)^2^** | ***P* value*** |
| --- | --- | --- | --- |
|  |  |  |  |
|  |  |  |  |
| Age (years) |  |  | 0.836 |
| <60 | 132 (58.7%) | 72.7 (3.7) |  |
| ≥60 | 93 (41.3%) | 72.3 (4.2) |  |
| Gender |  |  | 0.376 |
| Male | 131 (58.2%) | 74.7 (3.6) |  |
| Female | 94 (41.8%) | 69.4 (4.3) |  |
| BMI, (Kg/m^2^) |  |  | 0.823 |
| <24.00 | 132 (58.7%) | 72.6 (3.6) |  |
| ≥24.00 | 93 (41.3%) | 71.0 (4.5) |  |
| Location of primary tumor |  |  | 0.996 |
| Colon | 74 (32.9%) | 71.8 (5.0) |  |
| Rectum | 151 (67.1%) | 72.7 (3.3) |  |
| Pathological type |  |  | 0.014 |
| Protrude type | 143 (63.3%) | 78.4 (3.5) |  |
| Infiltrating or ulcerative type | 82 (36.7%) | 63.6 (4.7) |  |
| Degree of differentiation^1^ |  |  | 0.111 |
| High and Medium | 182 (80.7%) | 75.5 (2.9) |  |
| Low | 37 (16.4%) | 62.3 (7.4) |  |
| Unknown | 6 (2.9%) | 48.5 (16.9) |  |
| Histological type |  |  | 0.714 |
| Adenocarcinoma | 170 (75.6%) | 72.0 (3.1) |  |
| Other types | 55 (24.4%) | 74.6 (5.6) |  |
| Duke's Stage |  |  | <0.001 |
| A | 26 (11.6%) | 91.3 (5.4) |  |
| B | 100 (44.4%) | 81.6 (3.7) |  |
| C | 80 (35.6%) | 65.4 (4.7) |  |
| D | 19 (8.4%) | 23.8 (4.7) |  |
| Anastomat on surgery^1^ |  |  | 0.003 |
| Yes | 160 (71.2%) | 76.0 (3.3) |  |
| No | 55 (24.4%) | 60.9 (5.4) |  |
| Unknown | 10 (4.4%) | 77.7 (13.5) |  |
| Chemotherapy |  |  | 0.8 |
| No | 133 (59.0%) | 72.7 (2.8) |  |
| Yes | 92 (41.0%) | 65.7 (15.8) |  |
| Radiotherapy |  |  | 0.559 |
| No | 217 (96.4%) | 71.7 (3.5) |  |
| Yes | 8 (3.6%) | 73.5 (4.3) |  |
| Tumor size (mm) |  |  | 0.053 |
| <70.3 | 112 (49.8%) | 80.7 (3.6) |  |
| ≥70.3 | 113 (50.2%) | 64.5 (4.1) |  |
| CEA level(ng/ml) |  |  | 0.007 |
| <5 | 97 (43.1%) | 80.2 (4.1) |  |
| ≥5 | 128 (56.9%) | 66.4 (3.7) |  |
| CA19-9 level(U/ml) |  |  | <0.001 |
| <37 | 158 (70.2%) | 82.9 (3.1) |  |
| ≥37 | 67 (29.8%) | 49.5 (4.7) |  |

^†^ The unknown group was excluded for tumor differentiation and anastomat on surgery in Cox analysis.

^*^*P* value＜0.05 was considered statistically significant.

**Table S7. Association between FAM156B methylation and CRC survival in subgroup analysis.**

| ***FAM156B***^†^ | | **CRC (n=225), n (%)** | **Survival rate(%)** | | **Median survival time (month)(SD)** | ***P**** | **Univariate analyses** | | **Multivariate analyses** | | **PS analyses** | |
| --- | --- | --- | --- | --- | --- | --- | --- | --- | --- | --- | --- | --- |
|  |  |  | **3-year** | **5-year** |  |  | **Crude HR (95%CI)** | ***P**** | **HR_adj_**^‡^ **(95%CI)** | ***P**** | **HR_ps-adj_**^§^ **(95% CI)** | ***P**** |
| Gender |  |  |  |  |  |  |  |  |  |  |  |  |
| Male | Hypo- | 117 (89.3%) | 68 | 6 | 74.6 (3.8) | 0.991 | 1.000 |  | 1.000 |  | 1.000 |  |
|  | Hyper- | 14 (10.7%) | 64 | 56 | 61.0 (6.6) |  | 0.995 (0.425-2.328) | 0.991 | 1.210 (0.483-3.036) | 0.684 | 1.065 (0.447-2.535) | 0.888 |
| Female | Hypo- | 85 (90.4%) | 62 | 53 | 68.3 (4.6) | 0.465 | 1.000 |  | 1.000 |  | 1.000 |  |
|  | Hyper- | 9 (9.6%) | 88 | 63 | 66.6 (5.9) |  | 0.654 (0.202-2.123) | 0.48 | 0.787 (0.215-2.881) | 0.717 | 1.592 (0.487-5.207) | 0.442 |
| Age |  |  |  |  |  |  |  |  |  |  |  |  |
| ≤60 | Hypo- | 120 (90.9%) | 63 | 56 | 71.1 (3.9) | 0.188 | 1.000 |  | 1.000 |  | 1.000 |  |
|  | Hyper- | 12 (9.1%) | 83 | 74 | 69.7 (5.2) |  | 0.468 (0.146-1.499) | 0.201 | 0.744 (0.224-2.476) | 0.63 | 2.290 (0.712-7.364) | 0.165 |
| >60 | Hypo- | 82 (88.2%) | 7 | 59 | 73.3 (4.4) | 0.408 | 1.000 |  | 1.000 |  | 1.000 |  |
|  | Hyper- | 11 (11.8%) | 6 | 4 | 55.0 (7.6) |  | 1.442 (0.604-3.442) | 0.41 | 1.327 (0.495-3.559) | 0.573 | 0.684 (0.276-1.699) | 0.413 |
| Location of primary tumor | |  |  |  |  |  |  |  |  |  |  |  |
| Colon | Hypo- | 70 (94.6%) | 66 | 56 | 72.1 (5.1) | 0.871 | 1.000 |  | 1.000 |  | 1.000 |  |
|  | Hyper- | 4 (5.4%) | 75 | 5 | 55.2 (12.6) |  | 1.125 (0.268-4.716) | 0.872 | 0.593 (0.122-2.873) | 0.516 | 1.097 (0.223-5.393) | 0.909 |
| Rectum | Hypo- | 132 (87.4%) | 66 | 58 | 71.9 (3.6) | 0.553 | 1.000 |  | 1.000 |  | 1.000 |  |
|  | Hyper- | 19 (12.6%) | 72 | 6 | 64.8 (5.0) |  | 0.793 (0.361-1.740) | 0.562 | 1.094 (0.477-2.511) | 0.832 | 1.297 (0.589-2.855) | 0.519 |
| Duke's stage | | | |  |  |  |  |  |  |  |  |  |
| A+B | Hypo- | 115 (91.3%) | 81 | 71 | 84.2 (3.4) | 0.805 | 1.000 |  | 1.000 |  | 1.000 |  |
|  | Hyper- | 11 (8.7%) | 82 | 64 | 67.5 (5.5) |  | 1.147 (0.406-3.242) | 0.796 | 0.850 (0.267-2.699) | 0.781 | 0.959 (0.331-2.780) | 0.938 |
| C+D | Hypo- | 87 (87.9%) | 46 | 39 | 56.2 (4.5) | 0.275 | 1.000 |  | 1.000 |  | 1.000 |  |
|  | Hyper- | 12 (12.1%) | 63 | 53 | 58.0 (7.5) |  | 0.605 (0.242-1.515) | 0.283 | 1.006 (0.385-2.626) | 0.991 | 1.755 (0.691-4.458) | 0.237 |

^†^ The cutoffs of FAM156B determined by ROC curve were: 5%.

^‡^ HR_adj_ for age, gender, Duke's Stage, anastomat on surgery, and preoperative CA19-9 level in multivariate Cox regression analysis.

^§^HR_PS-adj_for PS as a covariate in multi-Cox analysis.

^*^*P*＜0.05 was considered statistically significant.

**Table S8. Association between PPP1R3F methylation and CRC survival in subgroup analysis.**

| ***PPP1R3F***^†^ | | **CRC (n=225), n (%)** | **Survival rate(%)** | | **Median survival time (month)(SD)** | ***P**** | **Univariate analyses** | | **Multivariate analyses** | | **PS analyses** | |
| --- | --- | --- | --- | --- | --- | --- | --- | --- | --- | --- | --- | --- |
|  |  |  | **3-year** | **5-year** |  |  | **Crude HR (95%CI)** | ***P**** | **HR_adj_**^‡^ **(95%CI)** | ***P**** | **HR_ps-adj_**^§^**(95% CI)** | ***P**** |
| Gender |  |  |  |  |  |  |  |  |  |  |  |  |
| Male | Hypo- | 52 (39.7%) | 7 | 57 | 72.8 (5.4) | 0.477 | 1.000 |  | 1.000 |  | 1.000 |  |
|  | Hyper- | 79 (60.3%) | 66 | 62 | 76.3 (4.7) |  | 0.827 (0.488-1.401) | 0.48 | 0.803 (0.455-1.418) | 0.449 | 1.067 (0.606-1.881) | 0.821 |
| Female | Hypo- | 36 (38.3%) | 55 | 49 | 62.0 (6.4) | 0.127 | 1.000 |  | 1.000 |  | 1.000 |  |
|  | Hyper- | 58 (61.7%) | 71 | 57 | 74.8 (5.4) |  | 0.638 (0.355-1.146) | 0.133 | 0.985 (0.507-1.914) | 0.965 | 1.312 (0.697-2.470) | 0.399 |
| Age |  |  |  |  |  |  |  |  |  |  |  |  |
| ≤60 | Hypo- | 53 (40.2%) | 55 | 46 | 62.7 (5.6) | 0.017 | 1.000 |  | 1.000 |  | 1.000 |  |
|  | Hyper- | 79 (59.8%) | 71 | 65 | 79.4 (4.6) |  | 0.539 (0.32-0.907) | 0.02 | 0.603 (0.341-1.067) | 0.082 | 1.428 (0.801-2.545) | 0.226 |
| >60 | Hypo- | 35 (37.6%) | 77 | 64 | 76.0 (6.1) | 0.637 | 1.000 |  | 1.000 |  | 1.000 |  |
|  | Hyper- | 58 (62.4%) | 64 | 52 | 69.7 (5.3) |  | 1.157 (0.629-2.128) | 0.64 | 1.509 (0.760-2.993) | 0.239 | 0.840 (0.447-1.577) | 0.587 |
| Location of primary tumor | |  |  |  |  |  |  |  |  |  |  |  |
| Colon | Hypo- | 29 (39.2%) | 65 | 5 | 64.8 (7.4) | 0.262 | 1.000 |  | 1.000 |  | 1.000 |  |
|  | Hyper- | 45 (60.8%) | 67 | 59 | 75.0 (6.4) |  | 0.679 (0.342-1.347) | 0.268 | 0.786 (0.363-1.702) | 0.54 | 1.323 (0.651-2.687) | 0.439 |
| Rectum | Hypo- | 59 (39.1%) | 63 | 55 | 69.7 (5.1) | 0.34 | 1.000 |  | 1.000 |  | 1.000 |  |
|  | Hyper- | 92 (60.9%) | 68 | 6 | 75.0 (4.5) |  | 0.794 (0.491-1.284) | 0.348 | 1.015 (0.597-1.728) | 0.955 | 1.056 (0.607-1.837) | 0.847 |
| Duke's stage | |  |  |  |  |  |  |  |  |  |  |  |
| A+B | Hypo- | 50 (39.7%) | 84 | 72 | 84.1 (4.9) | 0.865 | 1.000 |  | 1.000 |  | 1.000 |  |
|  | Hyper- | 76 (60.3%) | 79 | 68 | 83.7 (4.2) |  | 0.948 (0.514-1.748) | 0.864 | 1.088 (0.557-2.123) | 0.805 | 0.889 (0.451-1.753) | 0.733 |
| C+D | Hypo- | 38 (38.3%) | 39 | 29 | 47.1 (5.6) | 0.032 | 1.000 |  | 1.000 |  | 1.000 |  |
|  | Hyper- | 61 (61.7%) | 55 | 49 | 65.9 (5.7) |  | 0.578 (0.347-0.964) | 0.036 | 0.762 (0.439-1.322) | 0.334 | 1.585 (0.934-2.69) | 0.088 |

^†^The cutoffs of PPP1R3F determined by ROC curve were: 0%.

^‡^ HR_adj_ for age, gender, Duke's Stage, anastomat on surgery, and preoperative CA19-9 level in multivariate Cox regression analysis.

^§^ HR_PS-adjust_ for PS as a covariate in multi-Cox analysis.

^*^*P*＜0.05 was considered statistically significant.

**Table S9. Association between PIH1D3 methylation and CRC survival in subgroups.**

| ***PIH1D3*** ^†^ | | **CRC (n=225), n (%)** | **Survival rate(%)** | | **Median survival time (month)(SD)** | ***P**** | **Univariate analyses** | | **Multivariate analyses** | | **PS analyses** | |
| --- | --- | --- | --- | --- | --- | --- | --- | --- | --- | --- | --- | --- |
|  |  |  | **3-year** | **5-year** |  |  | **Crude HR (95%CI)** | ***P**** | **HR_adj_**^‡^ **(95%CI)** | ***P**** | **HR_ps-adj_**^§^**(95% CI)** | ***P**** |
| Gender |  |  |  |  |  |  |  |  |  |  |  |  |
| Male | Hypo- | 109 (83.2%) | 72 | 62 | 76.1 (3.8) | 0.386 | 1.000 |  | 1.000 |  | 1.000 |  |
|  | Hyper- | 22 (16.8%) | 49 | 49 | 65.6 (8.2) |  | 1.337 (0.690-2.590) | 0.389 | 1.153 (0.556-2.394) | 0.702 | 0.801 (0.41-1.568) | 0.518 |
| Female | Hypo- | 81 (86.2%) | 61 | 53 | 68.6 (4.6) | 0.648 | 1.000 |  | 1.000 |  | 1.000 |  |
|  | Hyper- | 13 (13.8%) | 84 | 59 | 61.7 (7.6) |  | 0.815 (0.319-2.080) | 0.669 | 1.241 (0.420-3.668) | 0.696 | 1.675 (0.626-4.476) | 0.304 |
| Age |  |  |  |  |  |  |  |  |  |  |  |  |
| ≤60 | Hypo- | 112 (84.8%) | 65 | 58 | 72.9 (4.0) | 0.878 | 1.000 |  | 1.000 |  | 1.000 |  |
|  | Hyper- | 20 (15.2%) | 59 | 54 | 59.0 (6.3) |  | 1.057 (0.518-2.159) | 0.879 | 1.006 (0.468-2.165) | 0.987 | 0.991 (0.483-2.036) | 0.981 |
| >60 | Hypo- | 78 (83.9%) | 7 | 58 | 73.1 (4.5) | 0.702 | 1.000 |  | 1.000 |  | 1.000 |  |
|  | Hyper- | 15 (16.1%) | 65 | 49 | 66.7 (10.3) |  | 1.172 (0.521-2.636) | 0.701 | 1.906 (0.811-4.478) | 0.139 | 1.021 (0.445-2.343) | 0.962 |
| Location of primary tumor | |  |  |  |  |  |  |  |  |  |  |  |
| Colon | Hypo- | 64 (86.5%) | 67 | 56 | 72.2 (5.2) | 0.894 | 1.000 |  | 1.000 |  | 1.000 |  |
|  | Hyper- | 10 (13.5%) | 67 | 53 | 56.8 (10.3) |  | 1.073 (0.376-3.063) | 0.895 | 1.837 (0.603-5.597) | 0.284 | 1.207 (0.406-3.59) | 0.735 |
| Rectum | Hypo- | 126 (83.4%) | 68 | 59 | 73.3 (3.6) | 0.709 | 1.000 |  | 1.000 |  | 1.000 |  |
|  | Hyper- | 25 (16.6%) | 6 | 52 | 68.7 (7.5) |  | 1.129 (0.604-2.110) | 0.705 | 1.213 (0.626-2.353) | 0.567 | 0.936 (0.497-1.762) | 0.837 |
| Duke's stage | | | |  |  |  |  |  |  |  |  |  |
| A+B | Hypo- | 109 (86.5%) | 8 | 69 | 83.1 (3.5) | 0.419 | 1.000 |  | 1.000 |  | 1.000 |  |
|  | Hyper- | 17 (13.5%) | 88 | 75 | 87.3 (7.2) |  | 0.660 (0.235-1.854) | 0.43 | 0.676 (0.229-1.997) | 0.478 | 1.675 (0.59-4.757) | 0.333 |
| C+D | Hypo- | 81 (81.8%) | 51 | 43 | 59.3 (4.8) | 0.479 | 1.000 |  | 1.000 |  | 1.000 |  |
|  | Hyper- | 18 (18.2%) | 38 | 31 | 44.5 (6.5) |  | 1.256 (0.664-2.378) | 0.483 | 1.213 (0.626-2.353) | 0.567 | 0.865 (0.45-1.663) | 0.663 |

^†^ The cutoffs of PIH1D3 determined by ROC curve were: 10%.

^‡^ HR_adj_ for age, gender, Duke's Stage, anastomat on surgery, and preoperative CA19-9 level in multivariate Cox regression analysis.

^§^ HR_PS-adjust_ for PS as a covariate in multi-Cox analysis.

^*^*P*＜0.05 was considered statistically significant.

**Table S10. The associations between methylation of PPP1R3F and CRC risk in subgroup analyses in the validation dataset**

| **DNA Methylation** | | **Gender** | | | | | | | | | | | |
| --- | --- | --- | --- | --- | --- | --- | --- | --- | --- | --- | --- | --- | --- |
|  |  | **Male** | | | | | | **Female** | | | | | |
|  |  | **Case (%)(n=87)** | **Control**  **(%)(n=84)** | **Crude OR**  **（95% CI）** | ***P* value^*^** | **OR_adj_（95% CI）** | ***P* value^*^** | **Case (%)(n=79)** | **Control**  **(%)(n=340)** | **Crude OR（95% CI）** | ***P* value^*^** | **OR_adj_**  **（95% CI）** | ***P* value^*^** |
|  |  |  |  |  |  |  |  |  |  |  |  |  |  |
| *PPP1R3F* | hypomethylation | 84(96.552%) | 83(98.81%) | 1.000 |  | 1.000 |  | 12(15.19%) | 57(16.765%) | 1.000 |  | 1.000 |  |
|  | hypermethylation | 3(3.448%) | 1(1.19%) | 0.337(0.034-3.309) | 0.351 | 2.773(0.281-27.375) | 0.383 | 67(84.81%) | 283(83.235%) | 0.889(0.452-1.75) | 0.734 | 1.136(0.575-2.244) | 0.713 |
| *PPP-CpG islands* | hypomethylation | 84(96.552%) | 83(98.81%) | 1.000 |  | 1.000 |  | 8(10.127%) | 61(17.941%) | 1.000 |  | 1.000 |  |
|  | hypermethylation | 3(3.448%) | 1(1.19%) | 2.964(0.302-29.08) | 0.351 | 2.773(0.281-27.375) | 0.383 | 71(89.873%) | 279(82.059%) | 1.94(0.888-4.24) | 0.096 | 1.924(0.878-4.216) | 0.102 |
| **DNA Methylation** | | **age** | | | | | | | | | | | |
|  |  | **≤ 60** | | | | | | **>60** | | | | | |
|  |  | **Case**  **(%)(n=128)** | **Control**  **(%)(n=348)** | **Crude OR**  **（95% CI）** | ***P* value^*^** | **OR_adj_（95% CI）** | ***P* value^*^** | **Case (%)(n=38)** | **Control**  **(%)(n=76)** | **Crude OR（95% CI）** | ***P* value^*^** | **OR_adj_**  **（95% CI）** | ***P* value^*^** |
|  |  |  |  |  |  |  |  |  |  |  |  |  |  |
| *PPP1R3F* | hypomethylation | 77(60.156%) | 106(30.46%) | 1.000 |  | 1.000 |  | 19(50%) | 34(44.737%) | 1.000 |  | 1.000 |  |
|  | hypermethylation | 51(39.844%) | 242(69.54%) | 0.29(0.19-0.442) | <0.001 | **0.196(0.096-0.4)** | **<0.001** | 19(50%) | 42(55.263%) | 0.81(0.371-1.766) | 0.596 | 0.102(0.012-0.86) | 0.036 |
| *PPP-CpG islands* | hypomethylation | 72(56.25%) | 113(32.471%) | 1.000 |  | 1.000 |  | 20(52.632%) | 31(40.789%) | 1.000 |  | 1.000 |  |
|  | hypermethylation | 56(43.75%) | 235(67.529%) | 0.374(0.247-0.566) | <0.001 | **0.101(0.043-0.237)** | **<0.001** | 18(47.368%) | 45(59.211%) | 0.62(0.283-1.358) | 0.232 | 0.272(0.052-1.42) | 0.122 |

OR odds ratio; CI confidence interval; ^*^*P* value＜0.025 was considered statistically significant.

**Table S11. The associations between methylation of PIH1D3 and CRC risk in subgroup analyses.**

| **DNA Methylation** | | **Gender** | | | | | | | | | | | |
| --- | --- | --- | --- | --- | --- | --- | --- | --- | --- | --- | --- | --- | --- |
|  |  | **Male** | | | | | | **Female** | | | | | |
|  |  | **Case (%)(n=87)** | **Control**  **(%)(n=84)** | **Crude OR**  **（95% CI）** | ***P* value^*^** | **OR_adj_**  **（95% CI）** | ***P* value*** | **Case (%)(n=79)** | **Control**  **(%)(n=340)** | **Crude OR**  **（95% CI）** | ***P* value*** | **OR_adj_**  **（95% CI）** | ***P* value^*^** |
|  |  |  |  |  |  |  |  |  |  |  |  |  |  |
| *PIH1D3* | hypomethylation | 84(96.552%) | 83(98.81%) | 1.000 |  | 1.000 |  | 29(36.709%) | 99(29.118%) | 1.000 |  | 1.000 |  |
|  | hypermethylation | 3(3.448%) | 1(1.19%) | 2.964(0.302-29.08) | 0.351 | 2.773(0.281-27.375) | 0.383 | 50(63.291%) | 241(70.882%) | 2.929(0.298-28.733) | 0.356 | 0.663(0.394-1.117) | 0.122 |
| cg07896193 | hypomethylation | 84(96.552%) | 82(98.795%) | 1.000 |  | 1.000 |  | 18(22.785%) | 111(32.647%) | 1.000 |  | 1.000 |  |
|  | hypermethylation | 3(3.448%) | 1(1.205%) | 0.708(0.424-1.184) | 0.188 | 2.773(0.281-27.375) | 0.383 | 61(77.215%) | 229(67.353%) | 1.643(0.927-2.912) | 0.089 | 1.653(0.93-2.937) | 0.087 |
| **DNA Methylation** | | **age** | | | | | | | | | | | |
|  |  | **≤ 60** | | | | | | **>60** | | | | | |
|  |  | **Case (%)(n=128)** | **Control**  **(%)(n=348)** | **Crude OR**  **（95% CI）** | ***P* value*** | **OR_adj_**  **（95% CI）** | ***P* value^*^** | **Case (%)(n=38)** | **Control**  **(%)(n=76)** | **Crude OR**  **（95% CI）** | ***P***  **value^*^** | **OR_adj_**  **（95% CI）** | ***P* value^*^** |
|  |  |  |  |  |  |  |  |  |  |  |  |  |  |
| *PIH1D3* | hypomethylation | 89(69.531%) | 151(43.391%) | 1.000 |  | 1.000 |  | 24(63.158%) | 31(40.789%) | 1.000 |  | 1.000 |  |
|  | hypermethylation | 39(30.469%) | 197(56.609%) | 0.336(0.218-0.517) | <0.001 | 0.793(0.453-1.389) | 0.417 | 14(36.842%) | 45(59.211%) | 0.402(0.180-0.896) | 0.026 | 0.467(0.141-1.541) | 0.211 |
| cg07896193 | hypomethylation | 79(61.719%) | 154(44.253%) | 1.000 |  | 1.000 |  | 23(60.526%) | 39(52%) | 1.000 |  | 1.000 |  |
|  | hypermethylation | 49(38.281%) | 194(55.747%) | 0.492(0.325-0.745) | 0.001 | 1.777(0.939-3.362) | 0.077 | 15(39.474%) | 36(48%) | 0.707(0.320-1.561) | 0.390 | 1.500(0.470-4.783) | 0.493 |

OR odds ratio; CI confidence interval; ^*^*P* value＜0.025 was considered statistically significant.
